# The Influence of Nutrition in Alzheimer's Disease: Neuroinflammation and the Microbiome vs. Transmissible Prion

**DOI:** 10.3389/fnins.2021.677777

**Published:** 2021-08-20

**Authors:** Laura Bello-Corral, Leticia Sánchez-Valdeón, Inés Casado-Verdejo, Jesús Ángel Seco-Calvo, Jesús Antonio Fernández-Fernández, María Nélida Fernández-Martínez

**Affiliations:** ^1^Department of Nursing and Physical Therapy, University of León, León, Spain; ^2^Institute of Biomedicine, University of León, León, Spain; ^3^University of the Basque Country, Leioa, Spain; ^4^Department of Biomedical Sciences, IBIOMED, University of León, León, Spain

**Keywords:** nutrition, Alzheimer's disease, neuroinflammation, microbiome, prion

## Abstract

Alzheimer's disease (AD) is a primary, progressive, neurodegenerative disorder. Many risk factors for the development of AD have been investigated, including nutrition. Although it has been proven that nutrition plays a role in AD, the precise mechanisms through which nutrition exerts its influence remain undefined. The object of this study is to address this issue by elucidating some of the mechanisms through which nutrition interacts with AD. This work is a qualitative systematic bibliographic review of the current literature searchable on various available databases, including PubMed, Web of Science, and Google Scholar. Our evidence comprises 31 articles selected after a systematic search process. Patients suffering with AD present a characteristic microbiome that promotes changes in microglia generating a proinflammatory state. Many similarities exist between AD and prion diseases, both in terms of symptoms and in the molecular mechanisms of pathogenesis. Changes in the composition of the gut microbiome due to dietary habits could be one of the environmental factors affecting the development of AD; however, this is probably not the only factor. Similarly, the mechanism for self-propagation of beta-amyloid seen in AD is similar to that seen in prions.

## Introduction

Alzheimer's disease (AD) is a primary neurogenerative disease characterized by subtle initial symptoms, such as alterations in memory that worsen incrementally. Symptoms progress to include behavioral changes and, in the final stages of the disease, the patient becomes wholly dependent (Mosconi and McHugh, 2015). This disease represents ~ 50–75% of all dementia cases, making it the most common form of dementia (Pistollato et al., 2016; Europe, 2017).

The pathology of AD is defined by the presence of extracellular senile plaques and intracellular neurofibrillary tangles formed, respectively, by beta-amyloid peptides and tau-proteins (Bhardwaj et al., 2017). In addition to these two key neurological pathologies, other features of AD include synaptic loss, marked hippocampal and cortical gliosis, a prominent oxidative stress, and an accumulation of vascular amyloid plaques within the brain, all of which contribute to neurological deterioration (Perl, 2010). Cerebral atrophy and excessive neuroinflammation are additional aspects of the disease (Association, 2018).

Although the origin of AD is still unknown, genetic and environmental factors that affect the evolution of the disease are known (Fern and Ruiz-Gabarre, 2019), such as different eating patterns, among others (Joseph et al., 2009; Abate et al., 2017).

At present, it remains an incurable disease, although there are pharmacological treatments, but with a limited therapeutic range (Mosconi and McHugh, 2015). However, there are studies that explore new therapies that could slow the progression of AD. Examples of these are cell therapy, based on the use of pluripotent stem cells from mature somatic cells, which may be a possible route in the treatment of AD (Guarnieri et al., 2018). A neuroprotective effect of estrogens against the inflammatory response produced by cholinergic neurons has also been observed in AD (Sarchielli et al., 2020). Neurons affected in AD show significant dysfunction in mitochondria and in the mitophagy process. For this reason, various therapeutic routes directed at mitophagy are being carried out in preclinical studies, since they can have wide applications due to their vital role in the initiation and progression of various neurodegenerative diseases (Rai et al., 2020).

As mentioned above, there are different eating patterns. Thus, increasing the intake of fruits, vegetables and fish in the diet has been shown to be beneficial for brain health (Moore et al., 2018). Nutrition influences cognitive and neurobiological changes in older adults, while a healthy diet seems to tip the balance in favor of a healthier senescence and seems to reduce the neurodegenerative risk in pathologies such as AD and other dementia entities (Gardener and Rainey-Smith, 2018). That is why nutrition and dietary patterns are key factors in the clinical care of people with cognitive impairment, because some vitamins, minerals and micronutrients have important antioxidant, anti-inflammatory and free radical scavenging properties that can protect oxidative damage, neuroinflammation and subsequent cognitive impairment (Dominguez and Barbagallo, 2016).

On the other hand, it is also related to nutrition, the constant risk of weight loss and a worsening of the nutritional status that patients with AD present as the disease progresses (Vieira et al., 2014). In AD, there is damage to areas of the cerebral cortex involved in the control of intake, also producing an impairment in serotonergic, dopaminergic and adrenergic neurotransmission. These neurotransmitters are essential in the regulation of eating behavior, which is why there is a progressive loss of appetite and finally a suspension of intake, which leads to a significant decrease in nutritional intake (Cardoso et al., 2013). Weight loss and malnutrition can contribute to the increase and progression of cognitive decline, loss of independence for daily activities, institutionalization, and increased mortality (Vieira et al., 2014).

Among the most notable dietary patterns is the Mediterranean diet, characterized by a high intake of vegetables, fruits, legumes, fish, cereals, and unsaturated fat, mainly olive oil. The Mediterranean diet has been found to be beneficial in reducing neurodegenerative risks. There is a combination between the Mediterranean diet and the diet focused on curbing Hypertension (DASH), this union produces what we call the Intervention Diet for Neurodegenerative Delay (MIND), which has a neuroprotective effect (Gardener and Rainey-Smith, 2018; Brink Van Den et al., 2019). This diet is characterized by the consumption of natural foods of vegetable origin, specifically the increase in green leafy vegetables, and a decrease in the consumption of foods of animal origin and with a high content of saturated fat (Gardener and Rainey-Smith, 2018; Brink Van Den et al., 2019).

However, the precise nature of how these diets act on cerebral physiology remains to be determined, although it is postulated to be due to growth in neurite extension and synaptogenesis (Gardener and Rainey-Smith, 2018).

Two theories can shed light on the mechanisms by which nutrition is involved, namely: the human microbiome as an important contributing factor to nutritional status, health, and disease (Bhattacharjee and Lukiw, 2013); and the appearance of prion disorders as a result of dietary exposure (Da et al., 2011).

### The Inflammatory Theory of the Microbiota

There are various factors that affect the acquisition and maturation of the microbiota, some of them are the type of delivery (cesarean or vaginal), antibiotics, breastfeeding, diet or environmental exposure to microbes (Cox and Weiner, 2018). Diet is recognized as one of the factors with the most influence on the intestinal microbiota. There is evidence that nutritional patterns with high or low amounts of fiber and diets based on plants or animals rapidly and reproducibly modify the intestinal microbial composition (Heiss and Olofsson, 2018).

Recent studies have shown that certain pathologies such as inflammatory bowel disease, irritable bowel syndrome, gastrointestinal infections, allergic diseases, diabetes and metabolic syndrome are related to an altered microbiota and are associated with the state mood, behavior, and cognition (Agahi et al., 2018; Giau et al., 2018). At present, the gut microbiota is of great interest in relation to various neurodegenerative diseases (Leblhuber et al., 2018), including AD (Rosa et al., 2018) as well as prion diseases (D'Argenio and Sarnataro, 2019). It has been observed that both, the production of beta-amyloid peptide in AD and neuroinflammation have been related to the intestinal microbiota, also microbiome has been described to have a role in the activation of the microglia in prion diseases (D'Argenio and Sarnataro, 2019).

Neuroinflammation and oxidative stress are factors that have been affected by the intestinal microbiota (Agahi et al., 2018). The gut microbiota and the central nervous system (CNS) are connected through multiple bidirectional pathways involving neuronal, endocrine, and immune signaling (Cox and Weiner, 2018).

Recent studies indicate that the human microbiota could contribute to the regulation of various neurochemical and neurometabolic pathways through a series of complex networks involving the microbe-ta-host symbiosis that systematically connect the gastrointestinal system, skin, liver and other organs with the CNS (Bhattacharjee and Lukiw, 2013).

### Prion Theory

Prion proteins exist both in normal cellular form and in an infectious, abnormally folded form. Upon coming into contact with the normal isoform, malformed prions cause these to misfold, which produces a cascade effect leading to cellular death (Abbott, 2016). Thus, there exist a cellular prion protein (PrPC), a glycosylphosphatidylinositol (GPI)-anchored glycoprotein found on the surface of cells and expressed in mammalian tissues, primarily in the CNS, and, in addition, its conformationally modified isoform, termed “Scrapie” (PrPSc), which is the principal component of prions (Toni et al., 2017).

Prions are an aetiological agent for a rare set of neurodegenerative disorders known as transmissible spongiform encephalopathies (TSEs) or prion diseases (Toni et al., 2017). The pathophysiology and pathogenesis of these diseases is characterized by marked brain spongiform change due to vacuolation, neuronal loss, astrocytosis, and intracerebral accumulation of folded prion protein (Walker and Jucker, 2015).

Several experimental transmission routes have been investigated for prion disorders such as intra-ocular, intraventricular, intraperitoneal, intraspinal and subcutaneous injections, however, most prion diseases are contracted by ingestion of PrPSc (Da et al., 2011).

It has been shown that the intestine has a key role in the transmission of prion proteins (Walker and Jucker, 2015). One of the pathways by which PrPSc gains access the intestine involves the endocytic membranous cells, which aid the translocation of these proteins from the intestinal epithelium into the lymphoid tissues. From here, prions can pass into the follicular dendritic cells, allowing their transport to the mesenteric lymphatic ganglions. In this way, the prion proteins can reach the enteric nervous system (ENS) and ultimately enter the CNS (Da et al., 2011).

It has been observed that, after oral administration, prions can resist the digestion process and enter the intestinal epithelium, where they interact with dendritic cells or macrophages, being able to accumulate in follicular dendritic cells and from there move to the ENS (Heiss and Olofsson, 2018). Enterocytes are the main cell population of the intestinal epithelium due to their ability to endocyte pathogens, nutrients, and macromolecules. It has been proposed that these cells could be a prominent entry site for food prions (Da et al., 2011).

Recently, it was suggested that amyloidogenic proteins, characteristic of the pathology of AD, are able to self-replicate in a similar manner to prions (Ashe et al., 2013). It should be noted that the neuropathology of Creutzfeldt – Jacob disease (CJD) shows similarities with AD, since both show neuronal damage, mitochondrial abnormalities, and oxidative damage, and both show inflammatory changes led by brain microglial proliferation. The main difference is the evolution of the disease, which progresses more rapidly in CJD than AD (Bastian, 2017).

### Objective

There is multiple evidence that could suggest that nutrition is closely related to AD, but the state of the art is still unclear; thus, the objective of this work is to shed light on the mechanisms by which nutrition is linked to AD through a systematic literature review. These mechanisms may be related to either of two theories: the prion theory or the microbiota inflammation theory.

## Materials and Methods

**Figure 1:**
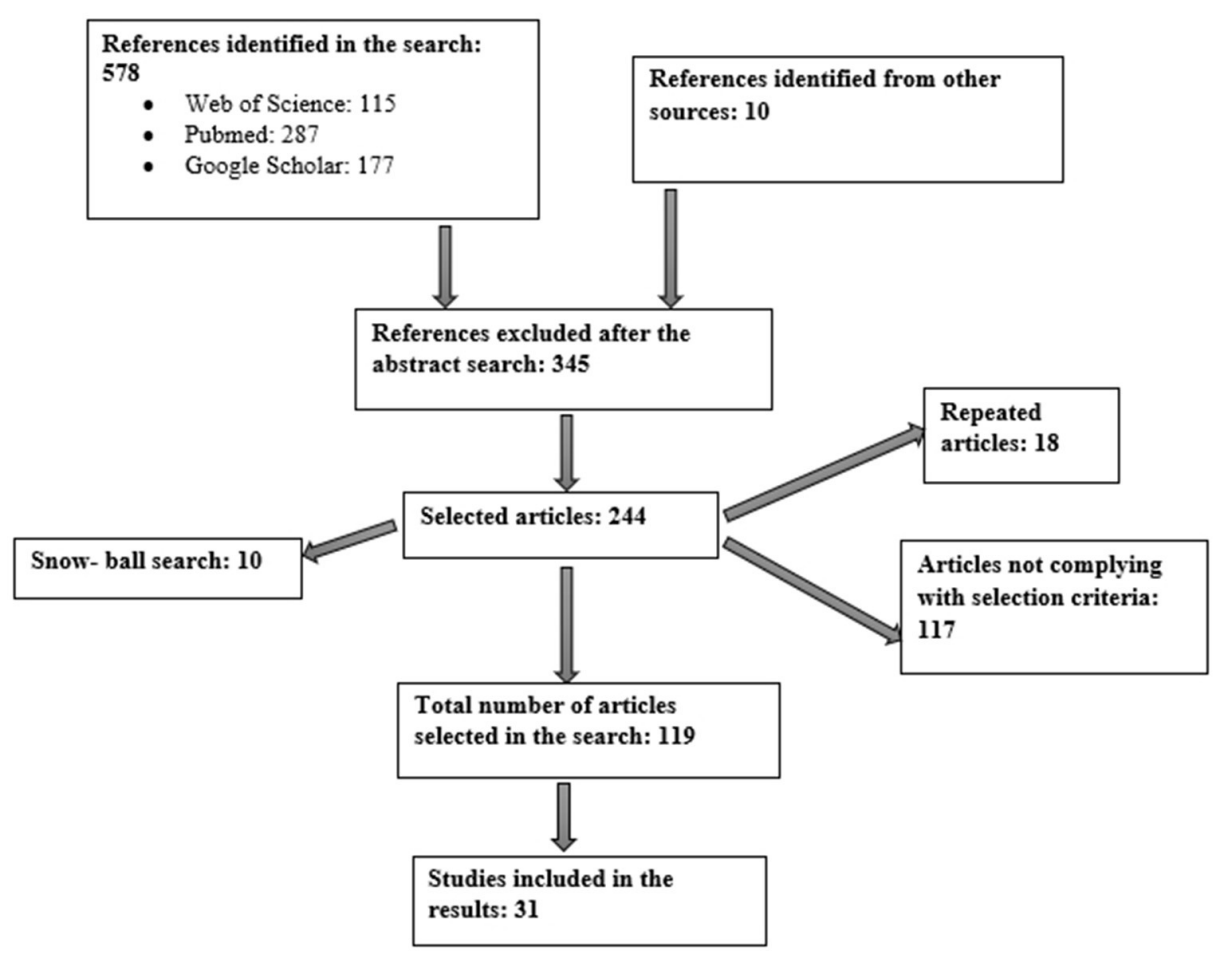
Flow diagram.

This work constitutes a qualitative systematic bibliographic review. The strategy employed for the literature search is summarized in Figure 1 and involved the use of several scientific databases, including Web of Science, Google Scholar and PubMed. The aim was to find answers to the questions posed in our objectives. Other sources of information were the publication catalogs in public libraries and various guides held by foundations; associations; and public and private institutions related to the themes addressed in the objectives of this work.

The methodology for our search strategy involved the use of certain key words, namely Alzheimer's disease, nutrition, neuroinflammation, gut microbiome, amyloid prion, transmissible prion.

The search was limited using filters to select articles in English published between 2009 and 2020 that focused on the themes pertinent to this study with the aim of uncovering evidence about the mechanisms through which nutrition is linked to AD.

After first reading all abstracts, we excluded those articles that did not contain material related to the theme of this work, that is, investigations into other sorts of dementia and those linking AD to forms of care other than nutritional.

In addition, through a “snowball effect” search of the literature reviews contained in the articles we consulted, we found other studies. Many of these are included here because they bring together some of the objectives set out in the present work.

## Results

The literature search yielded a total of 588 articles from the various databases and other information sources consulted. Of these, 244 were selected for full review and 31 were then chosen to form part of the evidence used in this work. Subsequently, the focus was on whether articles looked at the relationship between AD and nutrition as either mediated through the microbiota theory or the prion theory.

A total of 31 studies were selected, of which 11 are preclinical, carried out in mice with different age ranges (Morales et al., 2010; Stöhr et al., 2012; Pietri et al., 2013; Noh et al., 2014; Wu et al., 2014; Minter et al., 2016; Harach et al., 2017; Bonfili et al., 2018; Khan et al., 2018; Purro et al., 2018; Zhao et al., 2019) and other types of clinical investigations that included four cross-sectional studies (Claesson et al., 2012; Fernández-Navarro et al., 2016; Cattaneo et al., 2017; Vogt et al., 2017), two randomized, double-blind, placebo-controlled clinical trial (Akbari et al., 2016; Agahi et al., 2018), two prospective observational studies (Pendyala et al., 2012; Morris et al., 2016), one longitudinal study (Anastasiou et al., 2017), four clinical cases were also evaluated (Haraguchi et al., 2009; Yoshida et al., 2010; Jaunmuktane et al., 2015; Aoyagi et al., 2019) and seven were added systematic bibliographic reviews (Li et al., 2016; Lukiw, 2016; Bastian, 2017; Mancuso and Santangelo, 2017; Singh et al., 2017; Zhan et al., 2018; Dohrmann et al., 2019).

Of the 31 articles selected as evidence, 21 are included in the first section which concerns the microbiota and its links to AD. The remaining 10 articles are in the second section which outlines the prion theory in relation to AD.

As shown in Table 1, the first section is introduced by two studies describing the characteristic type of microbiota found in patients suffering from AD (Harach et al., 2017; Vogt et al., 2017). Later, studies were classified according to the form of analysis used in relation to their study of microbiota and AD. One study included centers on neuroinflammation in AD patients and its possible relation to microbiota characteristics (Cattaneo et al., 2017); see Table 2. Other studies, summarized in Table 3, highlighted the role of lipopolysaccharides (LPS) in the immune systems of people with AD (Noh et al., 2014; Lukiw, 2016; Zhao et al., 2019). Also included were two studies, simplified explanations of which can be found in Table 4, that analyzed the levels of gamma-aminobutyric acid (GABA), a neurotransmitter which appears to be important in AD (Wu et al., 2014; Li et al., 2016). In addition, a further five studies, explained in Table 5, were included because they deal with possible therapeutic approaches to AD (Akbari et al., 2016; Minter et al., 2016; Agahi et al., 2018; Bonfili et al., 2018; Khan et al., 2018). At the end of this section, a total of eight investigations were grouped together, see Table 6, in which microbiota were related with diet (Claesson et al., 2012; Pendyala et al., 2012; Fernández-Navarro et al., 2016; Hu et al., 2016; Morris et al., 2016; Anastasiou et al., 2017; Singh et al., 2017; Dohrmann et al., 2019).

**Table 1:** Microbiota and Alzheimer's disease (AD).

**Author**	**Year**	**Type of study**	**Objective**	**Method**	**Results**
Harach et al. (2017)	2017	Cross-sectional study	To evaluate the gut microbiota in transgenic mouse models with AD. To study the role of the microbiota in germ-free mice models	Eight-month-old AD transgenic mouse models and germ-free transgenic mice models	In transgenic mice with AD, a different microbiota was observed from the control group. In germ-free transgenic mice, beta-amyloid pathology was reduced
Vogt et al. (2017)	2017	Cross-sectional study	Characterization of the gut microbiota found in individuals with AD	A total of 119 participants were recruited from an Alzheimer's care center; of these 25 were diagnosed with AD dementia and 94 were not diagnosed with dementia	Participants with AD had a lower gut microbiota diversity and furthermore the bacteria found had a different taxonomic profile from that of the control group

**Table 2:** Microbiota and neuroinflammation.

**Author**	**Year**	**Type of study**	**Objective**	**Method**	**Results**
Cattaneo et al. (2017)	2017	Cross-sectional study	To investigate the possible role of the intestinal microbiota in the pathogenesis of AD by studying the association between the cerebral amyloidosis and the microbiota bacterial taxa having either pro or anti-inflammatory activity	A total of 83 patients were recruited: 10 cognitively healthy; 40 amyloid+ with cognitive deterioration and 33 amyloid– with cognitive deterioration	Amyloid+ subjects showed a lower abundance of *Eubacterium rectale* bacterial species and higher numbers of the pro-inflammatory species *Escherichia* and *Shigella*. Amyloid+ patients also had increased levels of pro-inflammatory cytokines in their blood

**Table 3:** Microbiota and the immune system.

**Author**	**Year**	**Type of study**	**Objective**	**Method**	**Results**
Noh et al. (2014)	2014	Preclinical study	To examine the regional distribution of inflammatory markers induced by LPS in young mice	Study involved 18 8-week-old ICR mice	Mice injected with LPS showed elevated levels of inflammatory markers in the frontal cortex and cerebellum. In addition, treated mice had increased numbers of microglia in the striatum, medial septum, frontal cortex, and hippocampus
Zhao et al. (2019)	2019	Preclinical study	To investigate the possible mechanisms of cognitive deterioration induced by LPS through an evaluation of the interaction between beta-amyloid peptides and neuroinflammation	Study involved 50 male C57BL/6J mice aged between 11 and 12 weeks	It was observed that administering LPS activated microglia and induced a loss of neuronal cells. The levels of pro-inflammatory cytokines were elevated in mice treated with LPS
Lukiw (2016)	2016	Bibliographic review	To highlight work done to study the potential contribution of factors originating in the microbiota, such as LPS, on the neuropathologic processes in AD	Authors completed a review of the literature concerning the effects of LPS in AD	Different bacterial species show slight differences in their response to inflammation Certain species associated with the microbiota can be regulated by diet

**Table 4:** Microbiota and gamma-aminobutyric acid (GABA).

**Author**	**Year**	**Type of study**	**Objective**	**Method**	**Results**
Li et al. (2016)	2016	Bibliographic review	To provide a general overview of the roles of beta-amyloid, tau-protein and APOE4 in GABAergic transmission and the possible modulation of GABAergic function as a therapy in AD	Authors carried out a review of the literature concerning the GABAergic system	For some time, it has been known that GABA receptors are resistant to AD. Recently evidence has come to light that the GABAergic system undergoes pathological changes and contributes to the development of AD
Wu et al. (2014)	2014	Preclinical study	To investigate the function of GABA in the dentate gyrus, a critical region of the brain involved in learning and memory	Study involved 5xFAD transgenic mice carrying mutated versions of APP and other proteins	A high GABA content was found in the reactive astrocytes of the dentate gyrus in a transgenic mouse model of AD. These findings are similar to those made regarding samples of brain tissue taken from patients with AD. The 5 × FAD mice showed symptoms of memory deficit

**Table 5:** Microbiota and therapy in AD.

**Author**	**Year**	**Type of study**	**Objective**	**Method**	**Results**
Akbari et al. (2016)	2016	Randomized double blind placebo controlled clinical study	To evaluate whether reinforcement of the gut microbiota by means of a probiotic supplement helps to reverse the cognitive, memory, and metabolic disturbances suffered by patients with AD	A total of 60 AD patients were recruited with ages ranging from 60–90 years. They were divided into two groups of 30 patients. One group took milk (control) while the other took a mixture of probiotics	After 12 weeks of the intervention there was an improvement in the MMSE scores of patients in the group taking probiotics in comparison with the control group. The differences between the groups were statistically significant
Bonfili et al. (2018)	2018	Preclinical study	To evaluate the potential of administering a formulation made of lactic acid bacteria and *Bifidobacteria* (SLAB51) to modulate the oxidative state in the brains of transgenic mice with AD	A transgenic mouse model for AD (3xTg-AD) was compared to a control group of wild mice	It was observed that the transgenic mice that received SLAB51 showed a reduction in levels of oxidative stress
Agahi et al. (2018)	2018	Randomized double blind placebo controlled clinical study	To evaluate the responsivity of inflammatory and oxidative biomarkers after treatment with probiotics	Subjects with AD aged between 65 and 90 years were randomly divided into two groups: 25 in the probiotics group and 23 in the placebo group	The group taking probiotics and the placebo group showed no differences in cognitive tests
Minter et al. (2016)	2016	Preclinical observational study	To examine the role of the microbiota in the regulation of amyloidosis in a mouse model	Study involved a murine model using 2-week-old mice. Subjects were divided into two groups: an antibiotics group and a control group	The changes in gut bacteria diversity produced due to long-term treatment with a combination regime of broad-spectrum antibiotics reduced the deposition of beta-amyloid plaques
Khan et al. (2018)	2018	Preclinical behavioral study	To use an adult mouse model to evaluate the neuroprotective effect of quercetin against the detrimental effects of LPS, such as neuroinflammation, synaptic disfunction and memory loss	A total of 45, 8-week-old mice were used. Subjects were divided into three groups (n = 15/group): a control group; one group given LPS; and another group given a combination of LPS + quercetin	Mice treated with quercetin showed significantly improved performance in memory tests compared to the group treated with LPS alone

**Table 6:** Microbiota and diet in AD.

**Author**	**YEAR**	**Type of study**	**Objective**	**Method**	**Results**
Morris et al. (2016)	2016	Prospective study	To look at the relationship between three types of diet, including the so-called Mediterranean diet, and the incidence of AD	Study involved a total of 923 participants between the ages of 58 and 98	Following the Mediterranean diet even to a limited extent can reduce the incidence of AD
Anastasiou et al. (2017)	2017	Longitudinal study	To make a comprehensive population study exploring the relationship between the Mediterranean diet and its key components, and specific areas of cognitive function	Study involved 1864 participants of which 90 were diagnosed with dementia and 223 were experiencing cognitive deterioration	Adherence to the Mediterranean diet was associated with better performance in terms of memory, language, visual-spatial awareness, and cognition
Dohrmann et al. (2019)	2019	Bibliographic review	To compare the effects of different dietary habits as either initiating or preventing senile dementia and AD	Authors completed a literature review	Both the Mediterranean and Japanese diets appear to be beneficial in AD. In contrast, the Argentine diet appears to be characterized by the consumption of foods that are damaging to brain health and may contribute to the development of AD
Hu et al. (2016)	2016	Bibliographic review	To analyse the possible relationship between AD and the gut microbiota	Authors completed a literature review, selecting articles based around the objectives of their study	Results overwhelmingly suggest that AD could begin in the intestine and is intricately related to imbalances in the gut microbiota
Fernández-Navarro et al. (2016)	2016	Cross-sectional study	To evaluate the association between adherence to the Mediterranean diet and its key components, and the fecal microbiota in a cohort of adults without any form of disease pathology	Sample comprised 31 subjects between 42 and 53 years of age, none of whom suffered from any pathologies	In subjects adhering most strictly to the Mediterranean diet, the study's findings showed that their microbiota contained the greatest proportion of *Bacteroidetes* (*p* = 0.001) and *Prevotellaceae* (*p* = 0.002) with a lower abundance of the phylum *Firmicutes* (*p* = 0.003) and bacteria from the *Lachnospiraceae* family (*p* = 0.045).
Singh et al. (2017)	2017	Bibliographic review	To evaluate the capacity of the host's diet to modulate intestinal bacteria, to understand how food choices affect human health by altering the gastrointestinal ecosystem	A systematic review of the literature was carried out in Medline including a total of 188 articles	In several studies an adherence to the western diet led to a marked reduction in the number of total bacteria and in the number of beneficial species such as *Bifidobacterium* and *Eubacterium*
Pendyala et al. (2012)	2012	Prospective observational study	Describe the metabolic effects of certain dietary patterns such as the Western diet	The study sample included 8 volunteer individuals between 55 and 66 years of age who followed the Western dietary pattern for 4 weeks	The Western-style dietary pattern induced significant increases (71%) in plasma endotoxin levels compared to a decrease (38%) following the intake of a prudent-style diet
Claesson et al. (2012)	2012	Cross- sectional study	To analyze the intestinal microbiota of the elderly and its association with diet and health	They included 178 sujects between 64 and 102 years old	A healthy diet in the elderly was correlated with a more diverse gut microbiota. It was analyzed that there is an association between the intestinal microbiota of elderly people and inflammation

In the second section are a collection of studies, shown in Table 7, that evaluate the resemblances between the pathogenic protein implicated in prion diseases and the beta-amyloid peptides characteristic of AD pathology (Aguzzi, 2014), in addition to the similarities between CJD and AD (Bastian, 2017). Other studies shown in this table analyzed the interaction occurring when pathological proteins and the beta-amyloid peptides found with AD are co-present in the brain (Morales et al., 2010; Stöhr et al., 2012; Aoyagi et al., 2019). A further set of investigations involved studies of the autopsies of patients that had died of CJD where deposits of beta-amyloid peptides were found, indicating possible iatrogenic transmission (Haraguchi et al., 2009; Yoshida et al., 2010; Jaunmuktane et al., 2015; Purro et al., 2018). The final study in this section looks at the enzyme 3-Phosphoinositide-dependent protein kinase-1 (PDK1), both in AD and prion disease (Pietri et al., 2013).

**Table 7:** Prion theory.

**Author**	**Year**	**Type of study**	**Objective**	**Method**	**Results**
Aguzzi (2014)	2014	Bibliographic review	To study a range of work where various forms of beta-amyloid peptides were found in association with AD, suggesting the hypothesis that this disease has much in common with prion diseases	Authors completed a review of recent literature	Prions are an abnormal version of the PrPC protein termed PrPSc which forms plaques of misfolded proteins. Its structure appears similar to that of amyloids
Stöhr et al. (2012)	2012	Preclinical study	To analyze the self-propagation abilities of beta-amyloids after injection into transgenic mice	The study involved a mouse model where APP23 and Tg(Gfap-luc) were crossed. CRND8 mice expressing mutated human APP were also used	Inoculation of APP23:Gfap-luc mice with brain homogenates containing beta-amyloids from the CRND8 mice, substantially elevated levels of beta-amyloid peptides in the APP23:Gfap-luc mice
Morales et al. (2010)	2010	Preclinical study	To analyze the interactions between the processes involved in the abnormal protein folding implicated in AD and prion diseases	Tg2576 AD mice were inoculated with PrPSc. A wild mouse model was used as a control	The onset of the clinical symptoms of prion disease was significantly more rapid in the Tg2576 AD mouse model than for animals in the control group
Haraguchi et al. (2009)	2009	Clinical case study	To document the results of an autopsy carried out on a known case of sporadic CJD, Lewy body disease, and AD	The patient was a 77-year-old man who died of pneumonia	The autopsy revealed deposits of CJD prion proteins in the cerebral cortex. Lewy bodies were seen in the cerebral cortex and the substantia nigra. In addition, senile plaques were found that were consistent with AD
Yoshida et al. (2010)	2010	Clinical case study	To analyze the results of an autopsy completed on a case of rare CJD with a codon mutation on the prion protein and AD pathology	The patient was a 77-year-old woman who died of pneumonia	The autopsy revealed abundant senile plaques and neurofibrillary tangles in areas of the neocortex. The prion protein associated with CJD was found in the hippocampus
Aoyagi et al. (2019)	2019	Clinical case studies	To describe the development of a cellular assay to compare levels of beta-amyloid and tau-proteins in cerebral tissue post-mortem	Sample comprised 75, recently deceased, AD patients	The beta-amyloid protein, which is similar to prions such as the pathological tau-protein, was found in elevated quantities in post-mortem brain tissue samples of AD patients. It was shown that there was an inverse relationship between the levels of pathological tau-protein found and the patient's age at death
Jaunmuktane et al. (2015)	2015	Clinical case studies	To analyze the results of autopsies completed on a set of subjects treated with growth hormone and who later died of iatrogenic CJD	Sample comprised 8 subjects between the ages of 36 and 51 at time of death	Beta-amyloid pathology was seen in the gray matter and blood vessels of the brains of four individuals. The beta-amyloid deposition in the gray matter was characteristic of AD
Purro et al. (2018)	2018	Preclinical study	Identify and analyze the growth hormone vials with which 8 subjects who died from CJD had been treated	They used mice between 6 and 8 weeks of age	Certain batches of growth hormone that patients were exposed to had substantial levels of beta-amyloid 40, beta-amyloid 42, and tau-protein
Bastian (2017)	2017	Bibliographic review	Investigate mixed cases of AD and CJD	Bibliographic review in which articles were selected based on their objectives	The neuropathology of CJD shows similarities to AD
Pietri et al. (2013)	2013	Bibliographic review	To analyze the mechanism of action of PDK1 in AD and prion diseases	They used 200 Tg2576 mice	It was observed that in neurons infected by pathogenic prions and in neurons of individuals with AD, the TACE protease was not present

## Discussion

The results of our review suggest that AD has been associated with dietary factors, because an excessive intake of saturated fat, among others, influence the disease. These dietary factors can promote or prevent the degeneration of nerve cells.

Numerous studies around the world have shown a correlation between various dietary patterns and their incidence in AD, indicating that diet acts as a modifiable risk factor (Barnard et al., 2014; Venturini et al., 2014; Ravi et al., 2018).

In a bibliographic review in which ecological and observational studies from various countries were selected to determine nutritional risk factors and AD, it was concluded that the Western dietary pattern characterized by a high consumption of meat is strongly associated with risk of developing AD. Traditional diets in India, Japan and Nigeria, with a very low intake of meat, were found to be associated with a 50% reduction in the risk of developing AD. Furthermore, this literature review shows that maintaining vitamin D concentrations above 75 nmol/L could help reduce the risk of AD (Grant and Grant, 2016).

### Microbiota and AD

One study observed that individuals with AD had less diversity and significant taxonomic differences in their gut microbiota compared to an age- and sex-matched control group. Similarly, their particular bacterial taxa were correlated with biomarkers for AD (Vogt et al., 2017). Findings showed that three specific bacterial genera were more abundant in subjects with AD and these correlated with the biomarkers for beta-amyloids and tau-protein (Vogt et al., 2017).

It was observed in another preclinical study that in the intestine of transgenic mice with AD there is a significant reduction in the bacteria that belong to the phyla *Firmicutes* and *Actinobacteria* and an increase in the number of *Bacteroidetes* and *Tenericutes* compared to the control group (Harach et al., 2017).

On the other hand, it has been shown that changes in the composition of the microbiota occur with age, which has an impact on protein function and function in the CNS (Amato et al., 2020).

#### Microbiota and Neuroinflammation

In a recent study of a clinical trial, subjects testing amyloid+ showed lower numbers of *Eubacterium rectale* bacteria and a greater abundance of *Escherichia* and *Shigella* bacteria in comparison to a control group (Cattaneo et al., 2017). In addition, it was observed that, compared to the control group, blood samples from amyloid+ patients showed increased levels of proinflammatory cytokines, interleukin 1 (IL-1) and IL-6, among others. Finally, a correlation was identified between increases in blood levels of proinflammatory cytokines and increases in numbers of the *Escherichia* and *Shigella* species of bacteria. The presence of the anti-inflammatory species, *Eubacterium rectale*, leads to an absence of proinflammatory cytokines (Cattaneo et al., 2017).

Recent studies have indicated that microbial dysbiosis can influence the formation of beta-amyloid fibrils, which could enhance neuroinflammatory activity in AD and the deposition of these beta-amyloid fibrils at brain level (D'Argenio and Sarnataro, 2019).

#### Microbiota and the Immune System

LPS are large molecules associated with certain pathogens which can activate an immune response in many types of cell, in particular those associated with the innate immune system. When activated these cells produce large quantities of proinflammatory cytokines, such as IL-6, IL-1α, and IL-1β, among others, which can cross the blood-brain barrier, reaching the brain via diffusion. Once inside the brain, cytokines act on the neuronal receptors and microglia, altering their activation state and physiology (Sampson et al., 2016).

Gram-negative bacteria are composed of an external membrane made of LPS, which constitutes their principal proinflammatory agent (Noh et al., 2014; Zhan et al., 2018). In rat studies, the administration of LPS resulted in neurotoxicity, microglial activation, and neuronal degeneration (Noh et al., 2014). Other authors have investigated the potential relationship between cognitive deterioration and neuroinflammation by administering LPS to mice (Noh et al., 2014). In these studies, LPS produced cognitive deterioration in the mice, accompanied by microglial activation and the loss of neurones from the hippocampus. Measurement of the levels of expression for several proinflammatory cytokines, such as tumor necrosis factor (TNF-α), IL-1β, prostaglandin E2 (PGE2), and nitric oxide (NO), revealed that levels of TNF-α and IL-1β were high compared to those measured for the control group. These findings demonstrate that LPS can provoke inflammation and the release of proinflammatory cytokines (Noh et al., 2014). The authors of this study presented these findings in a mouse model with neuronal inflammation and neurodegenerative pathology, as seen in AD (Noh et al., 2014).

Moreover, the authors of a review concluded that in the brain of patients with AD there is a significantly higher amount of LPS from large-negative batteries compared to the control group (Zhao et al., 2017).

However, a dual effect of TNF-α on the phenotypic plasticity of a model of cholinergic neuroblasts of the human basal forebrain has been studied, in which a negative effect on neurogenesis has been observed, but also a positive effect in differentiation, inducing phenotypic changes in neuroblasts still undifferentiated to a specific lineage (Guarnieri et al., 2020).

By comparison, LPS produced by the bacterium of the genus *Bacteroides fragilis* is recognized by microglia receptors in the same way as the proinflammatory 42 amino acid beta-amyloid peptide (Aβ42) associated with AD. This provides evidence that exposure of human brain cells to LPS from *B. fragilis* is a potent inducer of the proinflammatory transcription factor complex NF-Kb, a well-known trigger in the expression of the pathogenic pathways involved in neurodegenerative inflammation (Lukiw, 2016).

#### Microbiota and GABA

GABA is the principal inhibitory neurotransmitter found in the CNS of mammals (Li et al., 2016). Increased levels of GABA in the intestinal tract are correlated with raised levels of GABA in the CNS. Dysbiosis in the gut microbiota, particularly a reduction in numbers of *Lactobacillus* and *Bifidobacterium*, influences GABA production in the intestine and leads to a decrease in levels of this neurotransmitter in the CNS (Hu et al., 2016).

The first studies in the human brain post-mortem, and in animal models, concluded that both neurons and GABAergic receptors seem to be most resistant to AD pathology, showing only moderate losses with this disease (Li et al., 2016). Later post-mortem studies revealed a decrease in levels of this neurotransmitter within the frontal, temporal, and parietal cortices (Mancuso and Santangelo, 2017). Nevertheless, it has recently been shown that GABAergic neurotransmission undergoes severe pathological alterations in AD (Li et al., 2016). In fact, one study using an AD mouse model found high GABA levels in reactive astrocytes in the dentate gyrus. Reactive astrocytes are hypertrophic glial cells containing high levels of glial fibrillary acidic protein that are activated as a result of lesion or injury (Wu et al., 2014).

#### Microbiota and Therapy in AD

In one study the effects of probiotics on cognitive and metabolic function were investigated among patients with AD (Akbari et al., 2016). Compared to the control group, the study group taking a supplement of probiotics showed statistically significant improvements in their scores on the Minimental State Examination (MMSE): from 8.7 to 10.6 points (Akbari et al., 2016).

Taking a different approach, one preclinical study working with AD transgenic mice explored the properties of a probiotic mixture. The investigation demonstrated that probiotics noticeably reduced oxidative stress in the brains of AD transgenic mice (Bonfili et al., 2018).

However, other authors have concluded that for patients in the advanced stages of AD, taking probiotic supplements does not influence cognitive function or other biochemical factors (Agahi et al., 2018). This is despite the fact that previous work by the same authors using a different probiotic supplement showed that it had a favorable effect on cognition and other biochemical markers. In this way, not only are probiotic make-up and dose important but also the severity of disease and the time over which supplements are administered (Agahi et al., 2018).

In other work, it has been shown that antibiotics also induce changes in the gastrointestinal microbiota (Minter et al., 2016). It has been observed, in an AD mouse model, that long-term treatment with a combination regime of broad-spectrum antibiotics alters the composition and diversity of the intestinal microbiota and reduces the deposition of beta-amyloid plaques (Minter et al., 2016).

Finally, one investigation using adult rats inoculated with LPS showed that treatment with quercetin significantly reversed the synaptic loss induced by LPS in the cerebral cortex and the hippocampus, and improved the rats' performance in memory tasks (Khan et al., 2018).

#### Microbiota and Diet in AD

After a revision of multiple studies, it was discovered that the mechanisms that influence the onset of senile dementia and AD are associated with lowered levels of antioxidants and Omega-3 polyunsaturated fatty acids in the diet (Dohrmann et al., 2019). Consumption of Omega-3 fatty acids has a prophylactic effect against the neurotoxicity produced by AD, improves memory and learning, and increases neuronal protection. The longevity and high quality of life experienced by the Japanese is attributed to their diet. Likewise, the Mediterranean diet is recognized for its preventative benefits against certain chronic diseases and a reduction in the incidence of AD. These two dietary patterns ensure an intake of fatty acids and antioxidants at the same time as minimizing the intake of saturated fats from red meat. In contrast, the Argentine diet is characterized by a high consumption of processed and red meat, manufactured baked goods, and sugary drinks with a low intake of fish, fruits, and vegetables, all of which contribute to a higher risk of cognitive deterioration and AD (Dohrmann et al., 2019).

Dietary patterns containing a high level of fats tend to result in increased intestinal permeability and LPS absorption which, as a consequence, produce endotoxemia, systematic inflammation, and disease. By comparison, calorific restriction promotes health by optimizing the composition of the host microbiota by increasing the number of bacteria with positive health benefits, such as *Lactobacillus*, while reducing the number of bacteria malignant to the host (Mancuso and Santangelo, 2017).

One study looked at the effects of the Mediterranean diet on the microbiota (Fernández-Navarro et al., 2016). Findings showed that this diet is directly associated with a range of bacteria, such as *Bacteroidetes*, the Prevotellaceae family of bacteria and the *Prevotella* genus, and inversely related to the presence of bacteria of the *Firmicutes* phylum and *Ruminococcus* genus. Subjects who adhered most strongly to the Mediterranean diet had a higher proportion of *Bacteroidetes*, suggesting that this could be related to the greater consumption of carbohydrates, fiber, and vegetable proteins characteristic of this type of diet (Fernández-Navarro et al., 2016).

Thus, diet has been identified as a controllable environmental factor with a demonstrable influence on microbiota composition. One study completed with elders demonstrated that diet is associated with the microbiota to the degree that a healthy, varied diet promotes a more diverse gut microbiota (Claesson et al., 2012). Furthermore, evidence was uncovered for a connection between the intestinal microbiota and inflammation in elderly people (Claesson et al., 2012).

### Prion Theory and AD

The full structure of PrPSc is not yet fully understood, however, it appears to be similar to that of amyloids which, like PrPSc, are a family of filament-like structures with a tendency to form protein clusters (Aguzzi, 2014). Beta-amyloid peptide, hyperphosphorylated protein Tau and PrPSc have a tertiary structure rich in beta sheets, which promotes the self-assembly of monomers in small oligomeric species that have neurotoxic and fibrillar assemblages (Sarnataro, 2018).

One hypothesis, for which increasingly stronger evidence is being gathered, is that the deposits of beta-amyloids found in AD patients disperse progressively throughout the brain, suggesting a propagation mechanism similar to that of prions. In one study, transgenic mice were inoculated with brain homogenates containing beta-amyloid clusters which resulted in increased beta-amyloid plaque formation in the brains of test subjects (Stöhr et al., 2012). This led the authors to conclude that beta-amyloid clusters are indeed able to self-propagate and for this reason may be considered as prions (Stöhr et al., 2012).

The authors of a work indicated that there are multiple investigations that conclude that both beta-amyloid peptide and tau protein can be aggregated and disseminated to the brain by a mechanism similar to the prion (Sarnataro, 2018).

In another study, the objective was to analyze the interaction between the mis-folding processes in proteins implicated in AD, and prion diseases (Morales et al., 2010). When AD transgenic mice with amyloid plaques were inoculated with prions, the symptoms of prion disease were observed to emerge rapidly. In addition, the deposition of amyloid plaques increased significantly in mice infected with prions in comparison with the non-inoculated control group. The authors suggest that there is a strong interaction between AD and prionic pathologies, indicating that the process of mis-folding in the case of one protein can be a risk factor for the appearance of mis-folding in other proteins (Morales et al., 2010).

In 2009, a study was presented concerning the analysis of results from an autopsy completed on a patient with sporadic CJD, that is, without any genetic component (Haraguchi et al., 2009). An anatomopathological examination of brain tissue post-mortem showed neuronal loss, astrocytosis, and characteristic patterns of spongiosis disease appearing in patches throughout the cerebral cortex and lenticular nucleus. Deposits of prion proteins were found in the cerebral cortex. Lewy bodies were also discovered in the cerebral cortex and the substantia nigra, and senile plaques, compatible with AD, were observed in the neocortex. The authors concluded that this was an unusual case in that the probability of CJD appearing in conjunction with Lewy body dementia and AD is extremely low (Haraguchi et al., 2009).

However, a year later some of the same authors of the study outlined above published a second article in which they analyzed the results of another autopsy on a patient suffering from a form of CJD with a codon mutation on the prion protein (Yoshida et al., 2010). The anatomopathological brain post-mortem revealed severe spongiosis, neuronal loss, and astrocytic gliosis in the cerebral cortex. Furthermore, senile plaques and neurofibrillary tangles were seen in areas of the neocortex. The conclusions of this study were that the relationship between the deposition of prion proteins and the co-presentation of AD in this case was significant. The authors suggested that beta-amyloid proteins can act as a promoting factor for the deposition of prion proteins (Yoshida et al., 2010).

In a recent study, in which Prusiner himself collaborated, anatomopathological analysis of a group of subjects was undertaken to assess the levels of two proteins similar to prions: beta-amyloid and pathological tau-proteins (Aoyagi et al., 2019). It was shown that the amount of pathological tau-protein present was inversely related to patient longevity. The authors interpreted this correlation as an indication that a high level of “prion-like” tau leads to the most severe forms of AD and leads to a fatal outcome much earlier than in patients where the activity of “prion-like” tau is much lower (Aoyagi et al., 2019).

In 2015, a group of researchers published an article based on the study of the autopsies of eight individuals with iatrogenic CJD (iCJD) (Jaunmuktane et al., 2015). Subjects ranged in age from 36 to 51 years and had all been treated with cadaveric pituitary human growth hormone. Surprisingly, the brains of these individuals with iCJD contained beta-amyloid plaques in the gray matter and in the blood vessels. The deposition of beta-amyloid observed in the gray matter is typical of that found in AD, and that found in the blood vessels is characteristic of cerebral angiopathy and is not generally related to the prion activity seen in CJD (Jaunmuktane et al., 2015).

None of the patients had pathological mutations, such as apolipoprotein E-4 (APOE-4) or other hereditary alleles associated with a high risk of developing early onset AD. The authors hypothesized that the significant parenchymal and vascular deposits of beta-amyloid seen in the four younger patients with iCJD compared with other patients suffering with prion disease and control subjects from the general population suggests iatrogenic transmission of the beta-amyloid pathology in addition to the CJD agent is a result of the growth hormone treatment they received. This shows that healthy people exposed in this way are potentially at risk of iatrogenic AD and amyloid cerebral angiopathy (Vogt et al., 2017). This evidence is, nevertheless, only circumstantial and causality could not be concluded. For this reason, a further article was published in 2018 in which the authors demonstrated that batches of human growth hormone contained particles of beta-amyloid, which would be consistent with the theory of iatrogenic, human to human, transmission of beta-amyloid pathology. Thus, there is growing evidence that, at least under highly controlled laboratory conditions, beta-amyloid clusters can be transmitted in a manner similar to that seen with prions (Ashe et al., 2013).

Despite the diversity of symptoms, it has been observed that the neuropathology of CJD shares certain features in common with AD, such as the neuronal damage, mitochondrial anomalies, and oxidative damage. Both diseases cause inflammatory changes that take place due to generalized microglial proliferation in the brain. The principal difference seen is that sporadic CJD causes death within a few months, whereas AD is a disease that, in the majority of cases, has a prognosis of ~10 years life-expectancy (Bastian, 2017).

The authors of one study looked at the converting enzyme TNF-α (TACE), known principally for its role in mediating the elimination of TNF-α receptors (Pietri et al., 2013). These researchers observed that this enzyme was less commonly found in neurones infected with pathological prions than in the neurones of people with AD due to increased activity of the enzyme PDK1 in these neurones. The purpose of the enzyme PDK1 is to induce phosphorylation and caveolin-1 mediated endocytosis of TACE. Therefore, it is suggested that pharmacologic inhibition of PDK1 to relocate TACE, thus restoring its neuroprotective function, could be useful in both diseases (Pietri et al., 2013).

Finally, it should be mentioned that many neurodegenerative diseases caused by protein plaques, such as AD, Parkinson's disease, and TSEs, are generally caused by genetic mutations or are sporadic in nature. However, the possible horizontal transmission of these diseases could pose a distinct threat to public health. In this case, the gut would undoubtedly play an important role in propagating the prion proteins responsible for TSEs and would also have a significant part in any hypothetical transmissibility of AD given how similar these pathologies appear to be (Da et al., 2011).

### Recommendations for the Future

As future recommendations, we see interesting the implementation of diets that are adapted to AD. In these diets it would be remarkable if there were foods such as vegetables, fruit, whole grains, fish, nuts and dairy products with low fat content and a low percentage of candy, fried potatoes, processed meat, high saturated fat and butter.

We believe it would be interesting to look for a relationship between the proven increase in the prevalence of AD due to dietary changes with the appearance or increase in “prion-like” forms of beta-amyloids. This area is one that needs to be investigated further because there remain many unanswered questions and it could bring to bear significant findings that would contribute to our knowledge of the etiopathogenesis of this disease.

Another recommendation is to undertake a more in-depth evaluation of the therapeutic benefits to AD of controlled microbiota modification, either through fecal transplants, antibiotics, pro- and pre-biotic supplements, or diets specially designed with this pathology in mind.

## Conclusions

The main conclusion of our work is that, the etiopathogenesis of AD has been associated with dietary factors, since an excessive intake of saturated fats or a deficiency of vitamin E, among others, influence the disease. These dietary factors can promote or prevent the degeneration of nerve cells. Thus, diet is a relevant factor in cognitive functioning in general and in AD in particular, being able to delay or reduce the patient's symptoms.

The microbiota of patients with AD has a characteristic composition and this is linked to the formation of beta-amyloid peptides, increased systematic proinflammatory activity, and subsequent mental decline. Furthermore, dysbiosis of the microbiota results in changes in GABA levels which contribute to functional deficit in the brains of patients with AD. Diets low in Omega-3 polyunsaturated fatty acids and antioxidants provide lower resistance to neurotoxicity, and a high consumption of sugars and saturated facts provokes systematic inflammation that affects the CNS due to the changes they produce in microbiota composition in patients with AD. Changes in microbiota composition in the gut as a result of different dietary habits is probably one of the environmental factors that impacts the development of AD.

Furthermore, a key feature of AD is the presence of beta-amyloid clusters in the brain that form as a result of self-propagating, abnormally folded, beta-amyloid proteins. These mis-folded proteins are pathological in nature and are remarkably similar to prions, having a molecular propagation mechanism that is almost identical. Although spontaneous mutation is the most widely accepted hypothesis for the appearance of AD, the prion theory should now be considered as a possible cause of this disease.

## Data Availability Statement

The original contributions presented in the study are included in the article/supplementary material, further inquiries can be directed to the corresponding author/s.

## Author Contributions

LS-V and LB-C: conception and design. LS-V, LB-C, IC-V, and JS-C: data acquisition. JAF-F and NF-M: analysis and interpretation. LS-V, LB-C, and IC-V: writing of the article. LS-V, JS-C, and MNF-M: review of the article. All authors contributed to the article and approved the submitted version.

## Conflict of Interest

The authors declare that the research was conducted in the absence of any commercial or financial relationships that could be construed as a potential conflict of interest.

## Publisher's Note

All claims expressed in this article are solely those of the authors and do not necessarily represent those of their affiliated organizations, or those of the publisher, the editors and the reviewers. Any product that may be evaluated in this article, or claim that may be made by its manufacturer, is not guaranteed or endorsed by the publisher.
